# Case Report: Rapid recurrence of psoriasiform dermatitis upon sequential anti-PD-1 therapy with pembrolizumab and tislelizumab with 3-year follow-up

**DOI:** 10.3389/fimmu.2026.1760904

**Published:** 2026-03-04

**Authors:** Chao-Cheng Chi, Zi-Yun Li, Sui-Qing Cai, Zhuang-Li Tang

**Affiliations:** 1 Department of Dermatology, Xiamen Chang Gung Hospital, Ximan, China; 2 Department of Dermatology, Zhejiang University School of Medicine Second Affiliated Hospital, Hangzhou, China; 3 Department of Dermatology, Zhejiang University School of Medicine Affiliated Zhejiang Hospital, Hangzhou, China

**Keywords:** anti-PD-1 therapy, drug rechallenge, immune checkpoint inhibitors, immune-related adverse events, psoriasiform dermatitis

## Abstract

Psoriasiform eruption is an uncommon cutaneous immune-related adverse event (irAE) associated with anti-PD-1 therapy, and its rapid recurrence upon switching to a different anti-PD-1 agent is a scarcely documented phenomenon. We report the case of a 59-year-old man with stage IIB lung adenocarcinoma who developed a pruritic, scaly eruption after his fourth cycle of pembrolizumab. Histopathological examination confirmed a diagnosis of grade 2 psoriasiform dermatitis according to the Common Terminology Criteria for Adverse Events (CTCAE) version 5.0. The initial episode was resolved with systemic corticosteroids. However, upon switching anti-PD-1 therapy to tislelizumab, a similar but more widespread eruption recurred rapidly within a week. The recurrence was successfully managed with topical corticosteroid and antihistamines, and the anti-PD-1 therapy was subsequently discontinued. During the 3-year follow-up after discontinuation, the patient’s skin lesions resolved completely with no recurrence, and no tumor progression was observed. The reduced latency of psoriasiform dermatitis recurrence upon anti-PD-1 inhibitor rechallenge suggests a memory T cell–driven immune response. It also highlights that such irAEs were observed with the two PD-1 inhibitors pembrolizumab and tislelizumab used in this case and can be effectively managed. In this case, tumor progression was not observed after treatment cessation, although causality cannot be inferred.

## Introduction

Immune checkpoint inhibitors (ICIs) have revolutionized the treatment of advanced cancers but are associated with a spectrum of immune-related adverse events (irAEs). Cutaneous irAEs are among the most frequent, with maculopapular rash and pruritus being the most common presentations (1). Psoriasiform eruptions represent a rare subset of these cutaneous toxicities, with an estimated incidence of 3% following PD-1/PD-L1 inhibition (2).

While most irAEs are manageable, certain patterns pose unique clinical challenges. Tislelizumab, an anti-PD-1 antibody with an engineered Fc domain designed to minimize FcγR binding, has been associated with psoriasiform eruptions in pharmacovigilance analyses, although its overall cutaneous toxicity spectrum may differ from that of earlier PD-1 inhibitors (2, 3). Here we present a case of rapid and widespread recurrence of psoriasiform dermatitis following the switch from pembrolizumab to tislelizumab in a patient with lung adenocarcinoma. The temporal pattern of the rapidly recurrent psoriasiform dermatitis suggests an immune response driven by long-lived memory T cells.

## Case description

We describe a 59-year-old man with stage IIB lung adenocarcinoma. The patient had a 30-pack-year smoking history and had quit smoking 2 years before diagnosis. He denied a history of alcohol abuse, drug allergies, chronic inflammatory diseases, or recent vaccinations. Additionally, he had not been taking any regular medications prior to being diagnosed with cancer, and had no personal or family history of psoriasis or other autoimmune diseases. He underwent a right upper lobectomy via video-assisted thoracoscopic surgery in May 2021, followed by four cycles of adjuvant pembrolizumab (200 mg, i.v. every 3 weeks).

Thirteen days following the fourth infusion of pembrolizumab, which corresponded to day 78 after the commencement of anti-PD-1 therapy, the patient developed pruritic, scaly, erythematous plaques on his limbs and trunk, predominantly on extensor surfaces, affecting approximately 10% of the Body Surface Area (BSA). Concurrent systemic symptoms including fever, arthralgia, myalgia, oral ulcers and ocular complaints were absent. Review of systems and laboratory investigations including complete blood routine, liver function tests and renal function tests were within normal limits, with no impairment of self-care. According to the Common Terminology Criteria for Adverse Events (CTCAE) version 5.0, this cutaneous adverse event was graded as Grade 2.

**Figure 1:**
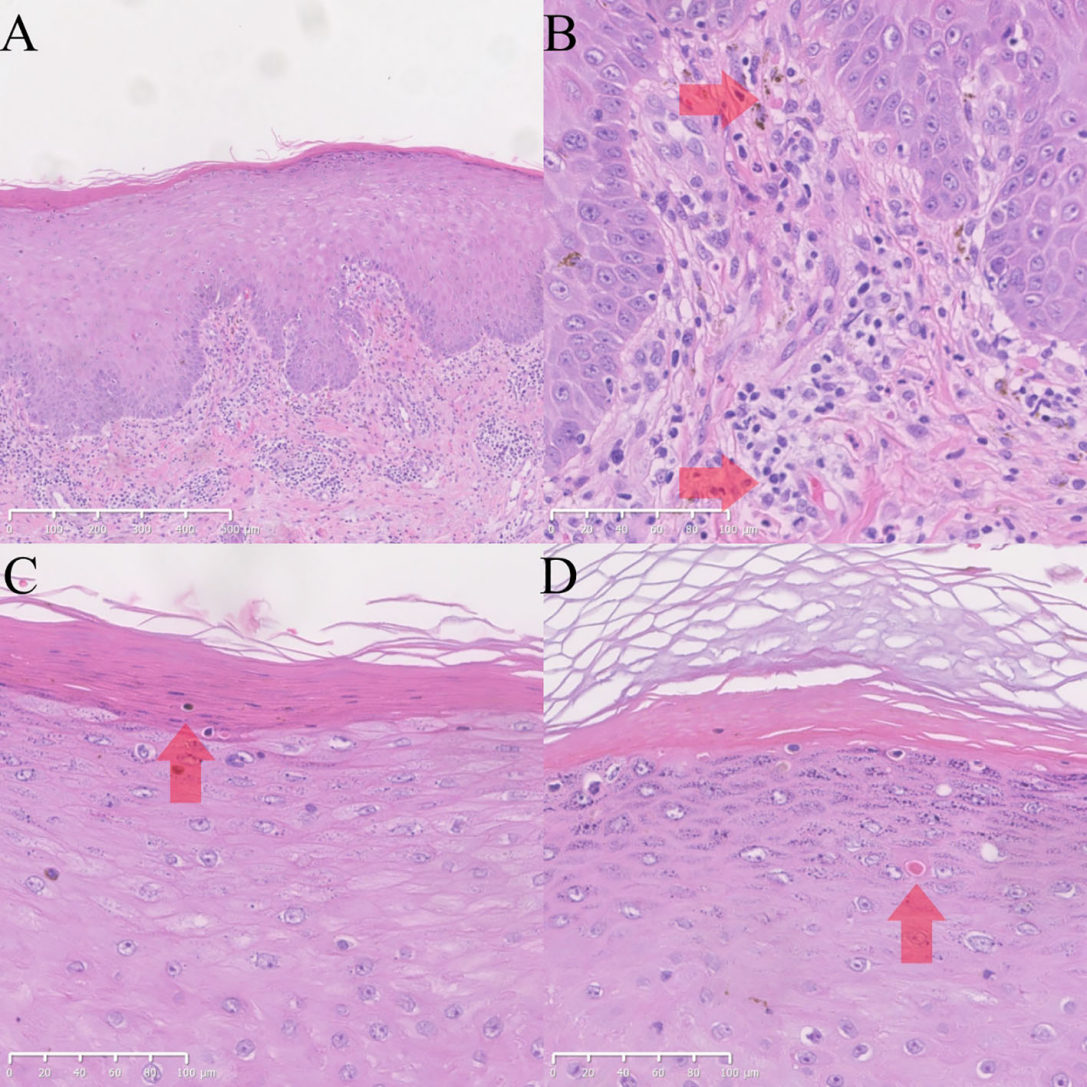
HE images of skin biopsy. **(A–D)**. HE images of the skin on the right calf. The submitted skin tissue measured 0.9 × 0.4 × 0.2 cm in size. There were irregular epidermal psoriasiform hyperplasia and inflammatory cell infiltration in the superficial dermis; the infiltrate is predominantly composed of lymphocytes intermingled with a small number of eosinophils (**(A)** magnification ×50; **(B)** magnification ×200). High-power magnification reveals focal parakeratosis in the stratum corneum (**(C)** magnification ×200). Within the stratum spinosum, there are focal dyskeratotic cells, which are characterized by homogeneous eosinophilic cytoplasm and pyknotic, hyperchromatic nuclei (**(D)** magnification ×200).

A skin biopsy was performed; histopathological examination revealed irregular epidermal psoriasiform hyperplasia, focal parakeratosis, and superficial dermal inflammatory cell infiltration consisting predominantly of lymphocytes admixed with a few eosinophils, as well as chronic spongiotic dermatitis with granular layer thickening and mixed inflammation (**Figures 1A–D**). Classic psoriatic features including Munro microabscesses and uniform rete ridge elongation were absent, consistent with the heterogeneous spectrum of ICI-related cutaneous immune-related adverse events.

The patient was initially treated with a 4-day course of intravenous methylprednisolone (40 mg daily), and concurrently received one-week courses of both intravenous glycyrrhizinate (120 mg daily) and sodium thiosulfate, alongside a 10-day course of topical mometasone furoate ointment applied twice daily. The eruption resolved completely after this one-week therapeutic period. In consultation with the oncology team, anti-PD-1 therapy was suspended. After a three-month treatment hiatus, the oncology team selected tislelizumab based on its engineered Fc domain and safety profile.

**Figure 2:**
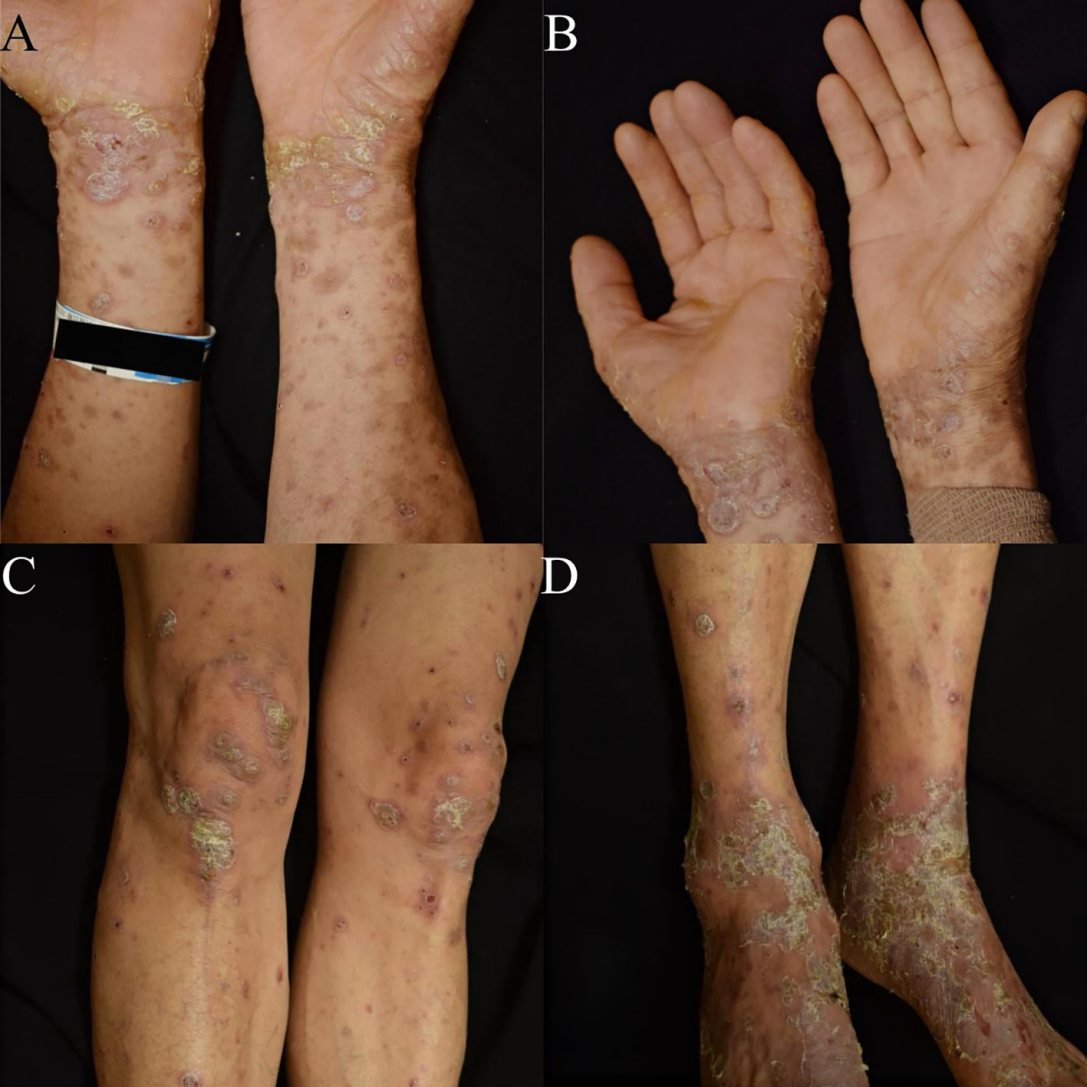
Clinical presentation of rapidly recurrent psoriasiform dermatitis following the first infusion of tislelizumab. **(A, B)** Well-demarcated erythematous plaques with fine micaceous scales on the flexor surfaces of the upper extremities. **(C, D)** Similar psoriasiform plaques involving the lower extremities; the knees and ankles show prominent erythema and scaling.

Notably, within one week of the first tislelizumab (200 mg, i.v.) infusion, a similar eruption, characterized by well-demarcated, erythematous plaques with micaceous scaling, recurred on his lower extremities and upper trunk, affecting approximately 12% of the BSA, with significant pruritus, also graded as CTCAE Grade 2. (**Figures 2A–D**). A repeat skin biopsy was proposed by the clinician but declined by the patient due to a strong personal preference. Given the patient’s refusal and the confirmatory histopathological findings from the initial biopsy, the repeat biopsy was not performed in clinical practice. The patient was successfully managed with intravenous glycyrrhizin (120 mg daily) as a corticosteroid-sparing agent, in combination with high-potency topical corticosteroid (halometasone ointment, twice daily), emollients, and oral ebastine (10 mg, three times daily). Pruritus resolved within 72 hours, and the erythematous plaques exhibited marked flattening with complete resolution of scaling within two weeks.

**Figure 3:**
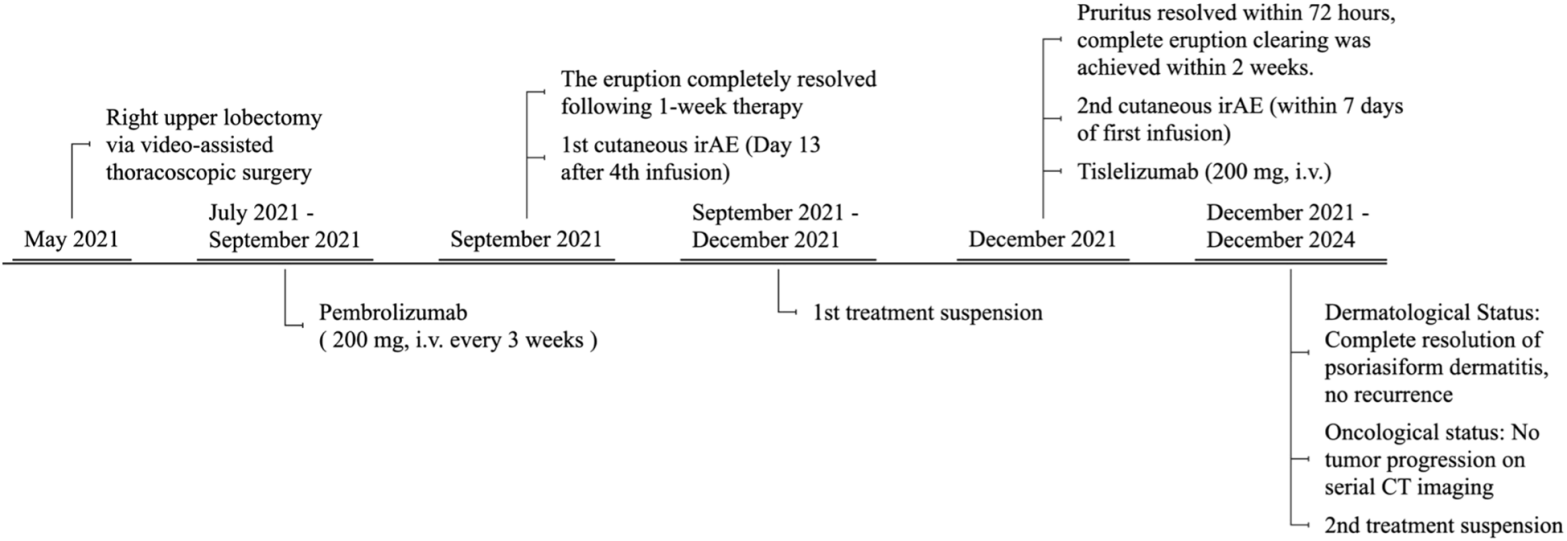
Timeline of PD-1 inhibitor-related psoriasiform dermatitis events during the treatment course of a lung adenocarcinoma patient.

Following this recurrence, anti-PD-1 therapy was permanently discontinued in consultation with the oncology team. Throughout the subsequent 3-year follow-up period, no recurrence of psoriasiform dermatitis or other significant cutaneous irAEs was observed on systemic skin examination. Concurrent surveillance with regular pulmonary CT scans showed no evidence of tumor recurrence (**Figure 3**).

## Discussion

This case illustrates a rapid and severe recurrence of psoriasiform dermatitis upon sequential use of two different anti-PD-1 antibodies, pembrolizumab and tislelizumab. Despite mixed inflammatory pathological changes, the core histopathological finding is irregular epidermal psoriasiform hyperplasia; clinically, the rash is characterized by sharply demarcated erythematous plaques with micaceous scaling. ICI-induced immune dysregulation often triggers mixed inflammatory infiltrates, leading to a non-strict one-to-one correspondence between clinical phenotype and pathological features, while core psoriasiform hyperplasia and typical clinical features confirm the diagnosis, and exclude other ICI-related rashes such as maculopapular rash and lichenoid dermatitis.

Our management approach was based on the clinical severity and progression rate of the eruption. According to the European recommendations by Apalla et al. (4), topical corticosteroids, vitamin D analogs, and other agents are preferred for Grade 1–2 psoriasiform irAEs. In the initial episode of this case, the rash progressed rapidly, involving 10% of the BSA with severe pruritus impairing daily life quality, making topical treatment inadequate for rapid control. Thus, systemic corticosteroids were administered, with glycyrrhizinate serving as a steroid-sparing agent to reduce the required steroid dosage, thereby mitigating potential steroid-related side effects. This approach aligns with individualized treatment and the guideline’s principle for significant symptoms for which topical therapy is inadequate, aiming to promptly relieve symptoms and ensure uninterrupted anticancer treatment.

According to the Naranjo Scale (5), the scores were 8 for pembrolizumab and 7 for tislelizumab. It has been reported that irAEs, regardless of severity, relapse in 34.2% of cases upon ICI rechallenge (6). Among these, the incidence of psoriasiform dermatitis is 4.1% with pembrolizumab and 4.8% with nivolumab (2, 7), while for tislelizumab, the incidence of immune-related rashes with monotherapy ranges from 10.7% to 13.5% (8), while psoriasiform dermatitis occurs infrequently, with documentation limited to case reports. A shared underlying mechanism may involve PD-1/PD-L1 inhibitors promoting T helper 17 (Th17) cell differentiation and activating pathways such as γδ low-expression T cells (9, 10).

The recurrence of psoriasiform dermatitis at previously affected sites is consistent with the concept of immunological disease memory, which has been attributed to tissue-resident memory T cells (TRMs) in previous studies (11). The initial exposure to pembrolizumab likely primed and established a population of autoreactive, skin-homing TRMs (12). These long-lived cells remain for a prolonged period in the skin during the treatment hiatus, creating a primed autoimmune milieu (13). In the context of anti-PD-1 immune checkpoint inhibitor therapy, although exogenous anti-PD-1 monoclonal antibodies may induce anti-drug antibody responses due to immunogenicity, this response is not the core mechanism driving therapeutic efficacy or immune-related adverse events. PD-1 blockade does not generate novel specific downstream targets; rather, it restores T-cell proliferation, cytokine secretion, and cytotoxic function by relieving the inhibitory signals mediated by the PD-1/PD-L1 pathway. The targets of memory T cells remain tumor-specific antigenic epitopes presented by MHC molecules on the surface of cancer cells or antigen-presenting cells. PD-1 antibody allows T cells to be reactivated against their intrinsic targets. This mechanism is also implicated in the development of immune-related adverse events. For instance, the underlying mechanism of anti-PD-1 therapy-induced psoriasis involves enhanced function of CD4+ and CD8+ T cells resulting from PD-1 blockade, which subsequently triggers immune imbalance (14, 15). A pivotal finding is the markedly shortened latency of psoriasiform dermatitis onset, from 13 days after the initial pembrolizumab administration (consistent with previous research reports (16)) to within one week after tislelizumab exposure, which could be explained by a memory T cell-mediated mechanism that memory T cells mount a more rapid attack upon re-encountering the antigen (13, 17). This observation raises the possibility that the “clinical resolution” of an irAE may represent symptomatic control rather than immunological eradication, potentially leaving behind a long-term “immunological scar” that could predispose patients to rapid irAE recurrence upon subsequent ICI exposure.

On a pharmacological level, although tislelizumab is engineered with a modified Fc domain to minimize Fcγ receptor (FcγR) binding and reduce antibody-dependent cellular phagocytosis (ADCP) of T cells (8), its core mechanism of action—PD-1 pathway blockade—remains fundamentally unchanged by this structural modification (18). This observation lends support to the hypothesis that the shared mechanism of PD-1 pathway blockade may be the dominant driver of this irAE. The mechanism is primarily linked to the disinhibition of pre-existing skin-specific T cells (including Th1, Th17, and CD8+ T cells) (19, 20) and the subsequent cascade of pro-inflammatory cytokines (e.g., IFN-γ, TNF-α, IL-17) (21); these factors collectively drive keratinocyte hyperproliferation and the development of a psoriasiform phenotype.

This phenomenon is not isolated to the skin. High recurrence rates upon ICI rechallenge have been documented for other irAEs affecting organs with robust resident immune networks, such as colitis and hepatitis (22), and the risk of thyroid toxicity is significantly higher with combination ICI therapy (23). This pattern further supports a unified model of TRM-driven, class-effect irAE recurrence.

A key strength of this case lies in its documentation of a rare psoriasiform dermatitis irAE following tislelizumab administration, coupled with long-term follow-up data: after discontinuing the ICI, the patient’s cutaneous adverse reactions resolved completely without long-term recurrence, while their malignant tumor remained in a progression-free state. This study has limitations inherent to a single-case report. The absence of molecular data to confirm the underlying T-cell-driven mechanism and the lack of a repeat biopsy for histopathological comparison are notable constraints.

## Conclusion

This case suggests that psoriasiform dermatitis can recur rapidly and severely upon sequential therapy with a different anti-PD-1 inhibitor, supporting the hypothesis of a class-effect potentially driven by memory T-cell responses rather than a drug-specific reaction. Critically, the eruption remained drug-dependent, resolving completely and without recurrence after therapy cessation. During the 3-year follow-up period, the patient’s lung adenocarcinoma remained stable without radiologic or serologic evidence of recurrence; however, this observation in a single case cannot establish a causal relationship between irAE management and oncologic outcome.

## Data Availability

The original contributions presented in the study are included in the article/supplementary material. Further inquiries can be directed to the corresponding authors.
